# Supplementary material for: The frequency limit of outer hair cell motility measured in vivo
Source: eLife. 2019 Sep 24;8:e47667. doi: 10.7554/eLife.47667 (PMC6759357; doi:10.7554/eLife.47667)
Supplement: Transparent reporting form [file elife-47667-transrepform.docx]

***eLife’s* transparent reporting form**

We encourage authors to provide detailed information *within their submission* to facilitate the interpretation and replication of experiments. Authors can upload supporting documentation to indicate the use of appropriate reporting guidelines for health-related research (see [EQUATOR Network](http://www.equator-network.org/%20)), life science research (see the [BioSharing Information Resource](https://biosharing.org/)), or the [ARRIVE guidelines](http://www.plosbiology.org/article/info:doi/10.1371/journal.pbio.1000412) for reporting work involving animal research. Where applicable, authors should refer to any relevant reporting standards documents in this form.

If you have any questions, please consult our Journal Policies and/or contact us: [editorial@elifesciences.org](mailto:editorial@elifesciences.org).

**Sample-size estimation**

- You should state whether an appropriate sample size was computed when the study was being designed
- You should state the statistical method of sample size computation and any required assumptions
- If no explicit power analysis was used, you should describe how you decided what sample (replicate) size (number) to use

Please outline where this information can be found within the submission (e.g., sections or figure legends), or explain why this information doesn’t apply to your submission:

No power analysis was used. The conclusions of the study are based on the observed patterns in the data (showing a low-pass characteristic) and their quantitative analysis (estimates of their corner frequencies from east-square fitting). This type of descriptive physiology does not lend itself to an experimental “design” in the statistical sense. The number of experimental animals included in the study was governed by our aim to cover the accessible frequency range (see Fig. 4) . The significance of each of the data points themselves (magnitudes and phases) was tested by a Rayleigh test (p=0.001). Only spectral components that passed this significance test were included in the analysis.

**Replicates**

- You should report how often each experiment was performed
- You should include a definition of biological versus technical replication
- The data obtained should be provided and sufficient information should be provided to indicate the number of independent biological and/or technical replicates
- If you encountered any outliers, you should describe how these were handled
- Criteria for exclusion/inclusion of data should be clearly stated
- High-throughput sequence data should be uploaded before submission, with a private link for reviewers provided (these are available from both GEO and ArrayExpress)

Please outline where this information can be found within the submission (e.g., sections or figure legends), or explain why this information doesn’t apply to your submission:

Number of experiments: Method Details. Technical replication is tested by the Rayleigh test; biological replication in the consistency across animals. Figure 4 contains the “raw data”, i.e., the distortion spectra measured in the Organ of Corti. Outliers: N/A. Exclusion/inclusion: see above remark Rayleigh test. Sequence data: N/A.

**Statistical reporting**

- Statistical analysis methods should be described and justified
- Raw data should be presented in figures whenever informative to do so (typically when N per group is less than 10)
- For each experiment, you should identify the statistical tests used, exact values of N, definitions of center, methods of multiple test correction, and dispersion and precision measures (e.g., mean, median, SD, SEM, confidence intervals; and, for the major substantive results, a measure of effect size (e.g., Pearson's r, Cohen's d)
- Report exact p-values wherever possible alongside the summary statistics and 95% confidence intervals. These should be reported for all key questions and not only when the p-value is less than 0.05.

Please outline where this information can be found within the submission (e.g., sections or figure legends), or explain why this information doesn’t apply to your submission:

Statistical methods and justification: Method Details, Basic Response Analysis and reference to Cooper et al 2018. Raw data: Fig. 4, see above remark. Apart from Rayleigh testing of the raw data (see previous items), statistical tests were not used; the results are descriptive rather than statistical.

(For large datasets, or papers with a very large number of statistical tests, you may upload a single table file with tests, Ns, etc., with reference to sections in the manuscript.)

**Group allocation**

- Indicate how samples were allocated into experimental groups (in the case of clinical studies, please specify allocation to treatment method); if randomization was used, please also state if restricted randomization was applied
- Indicate if masking was used during group allocation, data collection and/or data analysis

Please outline where this information can be found within the submission (e.g., sections or figure legends), or explain why this information doesn’t apply to your submission:

N/A. No groups were used.

**Additional data files (“source data”)**

- We encourage you to upload relevant additional data files, such as numerical data that are represented as a graph in a figure, or as a summary table
- Where provided, these should be in the most useful format, and they can be uploaded as “Source data” files linked to a main figure or table
- Include model definition files including the full list of parameters used
- Include code used for data analysis (e.g., R, MatLab)
- Avoid stating that data files are “available upon request”

Please indicate the figures or tables for which source data files have been provided:

Upon acceptance we will share the numerical data of all graphs using the FigShare platform (as we did previously, https://figshare.com/collections/Cooper_Vavakou_vanderHeijden/4147139).
